# Predictors of Mortality in Ruptured Abdominal Aortic Aneurysms

**DOI:** 10.7759/cureus.71024

**Published:** 2024-10-07

**Authors:** Dietrich V Jehle, Shayan Ravanassa, Micah K Browne, Blake Mireles, Krishna K Paul, Homar J Garza, Joshua Pevoto, Lauren G Bothwell, Mitchell W Cox

**Affiliations:** 1 Department of Emergency Medicine, University of Texas Medical Branch at Galveston, Galveston, USA; 2 Department of Vascular Surgery, University of Texas Medical Branch at Galveston, Galveston, USA

**Keywords:** abdominal aortic aneurysm (aaa), mortality predictors, rupture, serum bicarbonate, systolic blood pressure

## Abstract

Introduction

The outcome of a ruptured abdominal aortic aneurysm (AAA) without any interventions is close to uniformly fatal. The Society for Vascular Surgery suggests a door-to-intervention time of less than 90 minutes in a patient with a ruptured AAA. Admission factors associated with poor outcomes in ruptured AAAs include hypotension, renal insufficiency, severe anemia, advanced age, and cardiac arrest. Patients who are particularly at high risk for open AAA repair may be candidates for endovascular repair, which may decrease mortality. This study aimed to assess the relationship between systolic blood pressure (SBP) and serum bicarbonate levels in predicting mortality in patients with ruptured AAAs.

Methods

This retrospective study was performed using the United States Collaborative Network of 57 academic medical centers/healthcare organizations in the TriNetX database. A total of 4,226 patients with ruptured AAAs were identified. Patients were categorized based on SBP of ≤90 mmHg, any SBP, or >90 mmHg and further stratified by bicarbonate levels. Rounded cutoffs of the bicarbonate ranges (<10, 10.01-15; 15.01-20, >20.01) were chosen for interpretative purposes. Mortality outcome was assessed within 90 days after presentation for the ruptured AAA.

Results

After exclusions, 4,174 patients presented with ruptured AAA between September 30, 2003, and September 30, 2023, in the database. Overall, 90-day mortality in any SPB cohort was 28%. Patients who presented with a ruptured AAA with an SBP ≤ 90 had a 46.3% mortality. Those who presented with a SBP > 90 had a 20.1% mortality. Additionally, as bicarbonate levels decreased, mortality increased within each SBP group.

Conclusions

Early recognition and intervention are critical for survival in patients with ruptured AAAs. Metabolic acidosis is an important marker of the severity of hemorrhage in these patients. In this large cohort study of ruptured AAAs, mortality increases significantly with hypotension and metabolic acidosis, represented by lower bicarbonate levels. Abnormalities in the serum bicarbonate may be seen before severe changes in vital signs in hemorrhaging patients. Early recognition of metabolic acidosis may lead to earlier life-saving interventions in patients with ruptured AAAs.

## Introduction

Abdominal aortic aneurysm (AAA) rupture is a relatively common vascular disease complication and a major cause of cardiovascular mortality. AAAs are estimated to cause 1.3% of all deaths among men between the ages of 65 and 85 years in developed nations [1]. AAAs typically do not present with any symptoms until the point of rupture, which is associated with a 65% mortality rate [1]. Patients with an asymptomatic AAA are advised to monitor the progression of the diameter of the aneurysm through the use of ultrasonography at regular intervals every few months depending on various risk factors. If the aneurysm is at significant risk for rupture, the risks of surgery are favorable, and the patient’s estimated life expectancy indicates that surgery is a reasonable intervention. Some common surgical options include open surgical treatment or endovascular repair if the patient is a poor candidate for open surgery [1].

It is understood that rupture of an AAA occurs when the stress on the aortic wall exceeds the wall strength. Factors that might affect the strength of the aortic wall, and thus the likelihood of rupture, include biomechanical forces on the vessel wall and proteolytic degradation of the extracellular matrix of the vessel wall, among other variables [2,3]. Some of the more well-known risk factors associated with the development and eventual rupture of AAAs include tobacco use, advanced age, male sex, hypercholesterolemia (particularly those with lower high-density lipoprotein cholesterol), and family history [4]. More specific variables associated with mortality rates following surgical repair of an already-ruptured AAA include hypotension, renal failure, advanced age, and cardiac arrest [5]. Notably, serum creatinine greater than 2.0 mg/dL or systolic blood pressure (SBP) of less than 70 mmHg preoperatively in the context of either open or endovascular surgical repair of a ruptured AAA is considered strongly predictive of poorer postoperative outcomes and associated with greater morbidity and mortality, both in the short and long terms [6].

It is thought that the SBP being less than 70 mmHg at any point in time following the ruptured AAA and/or a serum creatinine above 2.0 mg/dL precipitates acute kidney injury, especially in the setting of surgical intervention, which further complicates postoperative recovery and may lead to worsening of outcomes [6,7]. Interestingly, a pilot randomized controlled study done in 2018 suggests that bolus high doses of sodium bicarbonate and hydration are promising preoperative interventions that can reduce endovascular aneurysm repair-related acute kidney injury (AKI), further underscoring the importance of serum bicarbonate levels concerning morbidity and mortality [7]. In an emergent setting, serum biomarkers can provide invaluable insights into overall patient morbidity and mortality. Metabolic acidosis and low serum bicarbonate are important markers of poor outcomes surrounding AAA rupture. Identification of metabolic acidosis can provide practitioners with a window of time before severe changes occur in the vital signs of a patient, which may lead to earlier life-saving interventions in those with a ruptured AAA [8-10]. Patients with larger preoperative anion gaps (AG) and lower bicarbonate levels have a poorer prognosis during open surgical repair of aortic aneurysms in an intensive care setting when compared to those with normal levels preoperatively [11].

The objective of this study was to evaluate the association between the patient’s SBP and serum bicarbonate levels in predicting mortality for ruptured AAAs. This study was presented at the American College of Emergency Physicians (ACEP) Research Forum on October 10, 2023, in Philadelphia.

## Materials and methods

This retrospective cohort study was conducted using TriNetX, a global federated health research network providing access to deidentified electronic medical records (EMRs), such as diagnoses, procedures, medications, laboratory values, and genomic information, from approximately 90 million patients in 57 large healthcare organizations (HCOs) within the United States. The information within the database was collected from HCOs including academic medical centers, specialty physician practices, and community hospitals. IRB approval was not required for this study, as all patient information utilized was deidentified. Patient data was obtained from the US Collaborative Network from September 30, 2003, to September 30, 2023. Patients were included in the study if they were diagnosed with ruptured AAAs as identified by the International Classification of Disease, 10th revision (ICD-10) code CM:I71.3. Three cohorts were created, which included patients with ruptured AAA and SBP ≤ 90, any SBP, and SBP > 90 using Observation Identifiers and Code (LOINC) 8480-6. The groups were further stratified based on varying bicarbonate levels (TNX:9021). Rounded cutoffs of the bicarbonate ranges (<10, 10.01-15; 15.01-20, >20.01) were chosen for ease of interpretation.

Outcomes

Mortality was assessed within 90 days after presentation for the ruptured AAA. Mortality data within the TriNetX platform is obtained from EMR data and HCOs, in conjunction with the national death registries. There is potential for missed death events when a patient is treated at an HCO not affiliated with the TriNetX network and subsequently experiences a fatal outcome outside of this network. However, this represents only a minor issue, as 94% of HCOs within the TriNetX network are also linked to the United States death registries. This percentage is steadily increasing as more HCOs continue to be linked with the registries. Patients were excluded if the index event occurred more than 20 years ago or had the outcome before arrival.

Statistical analysis

Univariate analysis was performed using the measure of association tool in TriNetX, which compares outcomes within the designated SBP and bicarbonate levels for each cohort reported as mortality risk. The final TriNetX data analyses were performed on September 30, 2023. The TriNetX platform provides access to aggregated counts and statistical summaries of deidentified patient records. Protected health information and personal data are anonymized and unavailable to platform users, and this project was deemed “to be exempt” by our Institutional Review Board.

## Results

A total of 4,226 patients (73% male, 26% female, mean age: 90 ± 11) were included who were diagnosed with a ruptured AAA, with blood pressure readings and bicarbonate levels recorded in their EMRs using the specified codes and period described. Demographics are shown in Table 1. After excluding patients who presented more than 20 years ago or with the outcome before arrival, 4,174 patients with ruptured AAA presented between September 30, 2003, and September 30, 2023, in the database.

**Table 1:** Patient demographics (n = 4,226) SD: Standard deviation.

	Mean	±SD
Age	78	±11
	Patients	% of cohort
Sex		
Male	3,085	73%
Female	1,099	26%
Unknown	42	1%
Ethnicity		
Not Hispanic or Latino	3,508	83%
Unknown ethnicity	634	15%
Hispanic or Latino	84	2%
Race		
White	3,296	78%
Unknown race	338	8%
Black or African American	380	9%
Asian	170	4%
Native Hawaiian or others	42	1%

**Figure 1:**
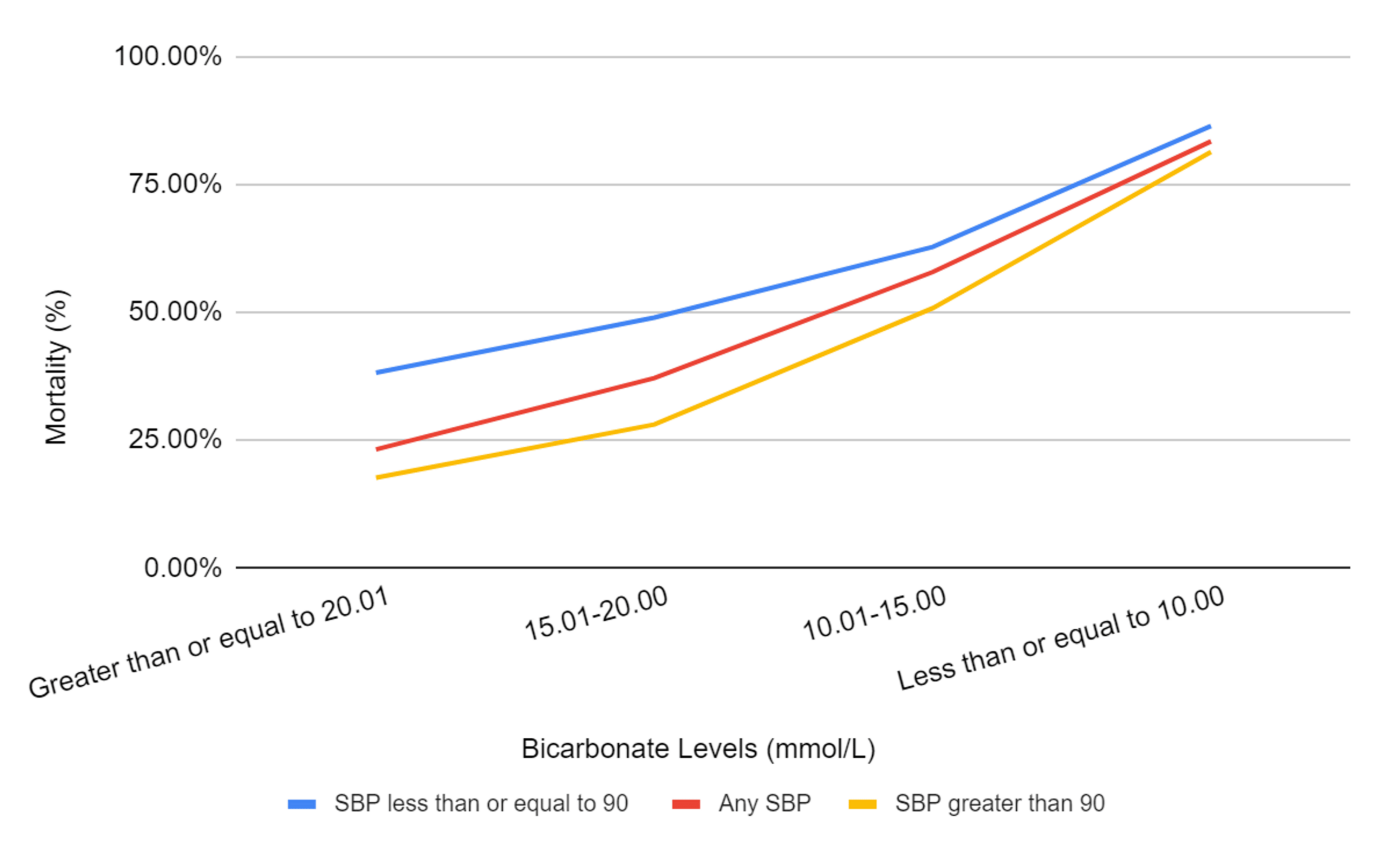
Outcomes of mortality versus bicarbonate in patients with rupture AAA and SBP cohorts AAA: Aortic abdominal aneurysm; SBP: Systolic blood pressure.

Among this cohort, the 90-day mortality was 28% (1,169 patients). Patients who presented with a ruptured AAA with an SBP ≤ 90 had a 46.3% mortality, while those who presented with an SBP > 90 had a 20.1% mortality on average. Notably, in each cohort of SBP, mortality increased as bicarbonate levels decreased. The three cohorts selected for bicarbonate levels with SBP ≤ 90, any SBP, and SBP > 90 are shown in Table 2 and Figure 1.

**Table 2:** Three-month mortality outcomes for AAA rupture patients at varying systolic blood pressure and bicarbonate levels SBP: Systolic blood pressure; AAA: Abdominal aortic aneurysm.

Cohort 1: AAA rupture patients with SBP less than or equal to 90			
Outcomes			
Bicarbonate levels (mmol/L)	Mortality (%)	Cohort size	Patients with outcomes
≥20.1	38.23%	1,007	385
15.01-20	49.02%	663	325
10.01-15	62.87%	272	171
= 10.0	86.54%	52	45
All	46.27%	1,301	602
Cohort 2: AAA rupture patients with any SBP			
Outcomes			
Bicarbonate levels (mmol/L)	Mortality (%)	Cohort size	Patients with outcomes
≥20.1	23.21%	3,589	833
15.01-20	37.19%	1,506	560
10.01-15	57.94%	447	259
= 10.0	83.54%	79	66
All	28.01%	4,174	1169
Cohort 3: AAA rupture patients with SBP greater than 90			
Outcomes			
Bicarbonate levels (mmol/L)	Mortality (%)	Cohort size	Patients with outcomes
≥20.1	17.70%	2,582	457
15.01-20	28.11%	843	237
10.01-15	50.86%	175	89
= 10.0	81.48%	27	22
All	20.05%	2,873	576

## Discussion

There was a significant association between mortality rates of ruptured AAAs and decreased serum bicarbonate levels within all blood pressure subgroups (SBP < 90, all SBPs, and SBP > 90) in this study. Decreased serum bicarbonate appears to be indicative of hemorrhage severity in these patients. Within each SBP cohort, patients with a ruptured AAA and serum bicarbonate levels of at least 20.1 mmol/L had the lowest mortality rates, while those with levels ≤ 10 mmol/L experienced the highest mortality rates. A stepwise increase in mortality rates with decreasing serum bicarbonate levels was noted across cohort stratifications.

Utilizing the TriNetX database, a substantial number of ruptured AAA patients were analyzed across three subgroups with varying SBP ranges, comparing mortality rates among different serum bicarbonate levels. This analysis, spanning from November 30, 2002, to November 30, 2022, represents the first attempt to establish a link between serum bicarbonate levels and mortality outcomes in patients with ruptured AAAs. The accessibility of serum bicarbonate as a laboratory measure is crucial to the early clinical decision-making for the management of patients with ruptured AAAs in both emergency and outpatient settings.

Previous studies have identified common risk factors for AAA development and rupture including AAA diameter, growth rate, age, gender, smoking status, hypertension, hypercholesterolemia, and dyslipidemia, among others [2-5]. Biomechanical factors such as aortic compliance and peak wall stress, along with aortic calcification, have been linked to increased rupture risk [2,3]. Increased calcification rates of the aorta may theoretically present a greater diffusion barrier for oxygen to be delivered to the outermost layers of the vessel, thus further weakening the integrity of the aorta and causing atrophy of the intimal and medial layers. Although studies on acid-base disorders in AAA prognosis are limited, findings from two notable studies underscore the association of metabolic acidosis and elevated AG with higher mortality rates in trauma and AAA surgical repair contexts, reinforcing the potential prognostic value of serum bicarbonate levels [10,11].

AAA repair is generally recommended for low-risk patients with aneurysms that are 5 cm or larger in diameter [12]. Timely screenings are crucial, especially for populations at higher risk for AAA. This is particularly important for elderly males, as the mortality rate can reach up to 90% after an aneurysm ruptures [13,14]. Effective management of AAA to predict and prevent rupture is essential, as repair procedures are safer and more effective than the risk of rupture. Although clinical examinations have limited effectiveness in detecting AAA, comprehensive physical exams remain necessary for at-risk groups [13,15]. Patients with AAA often experience acute abdominal and lower back pain, and any suspicion should prompt abdominal palpation, auscultation, and imaging [13]. Considering a patient's clinical history is also crucial, as symptoms are often mild until a rupture occurs.

This study's findings suggest that serum bicarbonate levels may be a more potent mortality predictor than blood pressure in ruptured AAA patients. The prompt availability of serum bicarbonate test results is crucial for early intervention. Despite potential limitations such as selection bias and unaccounted confounding variables, this research is a pivotal step toward understanding the role of serum bicarbonate in ruptured AAA outcomes. Future studies should focus on developing targeted interventions based on metabolic acidosis status to enhance patient survival prospects.

Limitations

This study acknowledges several limitations. This design precludes establishing causation, allowing only the inference of associations based on historical data. Selection bias may also be present due to the use of TriNetX for cohort selection based on ICD-10 disease classifications. The study's focus on specific factors, such as hypotension and bicarbonate levels, does not account for other potential confounding variables. Although smaller sample sizes are a limitation in the generalizability of studies evaluating AAA ruptures, this is one of the largest studies looking at ruptured AAAs, which strengthens the power of the results. Furthermore, the TriNetX privacy policy precludes assessing whether patient clustering occurred at specific centers due to the deidentified nature of the dataset. Another challenge involves the use of TriNetX, a large global research network, which restricts access to direct clinical patient scoring systems consistently used for outcome determination in aneurysm patients. The absence of predictive variables for this condition and overall health status may limit the accuracy and completeness of these predictions. Essential scoring systems for risk stratification, such as the Glasgow Aneurysm Score, Apache II Score, and the American Society of Anesthesiologists (ASA) score, are not fully available in the database, somewhat constraining our study's insights.

## Conclusions

This retrospective cohort study has shown that progressively lower levels of serum bicarbonate are associated with a significantly greater risk of mortality following a ruptured AAA regardless of blood pressure range. Due to the nature of ruptured AAAs, earlier intervention is essential to improving patient outcomes. Abnormalities in the serum bicarbonate levels can be seen before severe changes to a patient’s vital signs in a hemorrhagic patient. When attempting to control a ruptured AAA, every second matters, and our study aims to shift the paradigm toward earlier, proactive intervention in a patient’s disease course. Future research on the association between bicarbonate levels and mortality outcomes in ruptured AAA patients is highly encouraged, particularly with regard to the temporal relationship between decreasing bicarbonate levels and severe changes in vital signs in a hemorrhaging patient.
